# Spatiotemporal patterns of anticoagulant rodenticide exposure in bobcats across the northeastern United States

**DOI:** 10.1371/journal.pone.0360055

**Published:** 2026-10-08

**Authors:** Jennifer A. Grauer, David B. Needle, Krysten L. Schuler, Patrick J. Connelly, Gretchen Fowles, Erica A. Miller, Brehan Furfey, Kim Royar, Mandy B. Watson, Jacob DeBow, Jacqueline L. Frair

**Affiliations:** 1 Department of Environmental Biology, College of Environmental Science and Forestry, State University of New York, Syracuse, New York, United States of America; 2 New Hampshire Veterinary Diagnostic Laboratory, Department of Molecular, Cellular & Biomedical Sciences, College of Life Sciences and Agriculture, University of New Hampshire, Durham, New Hampshire, United States of America; 3 Wildlife Health Lab, Public and Ecosystem Health Department, College of Veterinary Medicine, Cornell University, Ithaca, New York, United States of America; 4 Office of Fish and Wildlife Health and Forensics, New Jersey Department of Environmental Protection – Fish and Wildlife, Hampton, New Jersey, United States of America; 5 Endangered and Nongame Species Program, New Jersey Department of Environmental Protection – Fish and Wildlife, LebanonNew Jersey, United States of America; 6 Vermont Fish and Wildlife Department, Springfield, Vermont, United States of America; 7 New York Department of Environmental Conservation, Albany, New York, United States of America; 8 New Hampshire Fish and Game Department, Lancaster, New Hampshire, United States of America; Universidade Federal de Minas Gerais, BRAZIL

## Abstract

Anticoagulant rodenticides are a pervasive threat to non-target wildlife health, with exposure in predators frequently driven by the interface of human development and wild spaces. Long-term regional data are needed to understand how exposure rates vary in relation to changing regulation and public use of rodenticide compounds. We analyzed opportunistic samples collected from 2013–2024 across the northeastern United States to quantify rates of anticoagulant rodenticide (AR) contamination in bobcats (*Lynx rufus*) and identify spatial hot spots of exposure. We detected seven AR compounds in 352 bobcats (59% exposed) across 4 states; the highest levels of exposure were in New Hampshire and Vermont. Our spatial clustering algorithm indicated localized hot spots of AR detection across New Hampshire, Vermont, and New Jersey, with New York clusters appearing cold by comparison. Exposed bobcats had 1–4 AR compounds (mean = 1.07, sd = 1.17) in their system and exposure rates varied by state, age, and sampling source (harvest versus roadkill) but not sex. Predicted odds of AR exposure increased by approximately 35%, and the predicted number of compounds increased by approximately 17%, for each year increase in bobcat age. Despite federal restrictions on the use of second-generation ARs going into effect during the course of our study, AR detection rates increased steadily over time at a rate of 25% per year. Our observations were similar to broad spatial patterns of AR exposure in northeastern fisher (*Pekania pennanti*), reflecting the ubiquity of AR exposure across predators with differing habitat use. This work provides baseline exposure information for a mesocarnivore species of interest, highlights a concerning trend of increasing AR exposure over time, and contributes to the growing evidence of widespread rodenticide contamination of non-target wildlife.

## Introduction

Environmental contaminants are pervasive stressors that pose a substantial and growing challenge for wildlife management [1]. Expanding development and encroachment into wildlands fragments habitat [2], increasingly places wildlife in conflict with humans [3], and exposes wildlife and their surroundings to deleterious contaminants [4]. Enacting effective governance to minimize harmful effects to biodiversity is fraught with difficulty [5], including long bureaucratic timelines and logistical limitations to enforcement, monitoring, and evaluation of success [6]. Yet, balancing considerations of wildlife health with human health and socioeconomic priorities is a persistent need. A prime example is rodent control using anticoagulant rodenticides, which directly and indirectly impacts the health of non-target wildlife [7].

Anticoagulant rodenticides (ARs) are used worldwide across agricultural, rural, suburban, and urban environments to kill rodent pests by inhibiting their blood-clotting ability and causing death by fatal hemorrhaging [8]. Non-target wildlife species are frequently exposed to and can bioaccumulate ARs via direct poisoning or consumption of contaminated prey [9], leading to both individual and potential population level effects [10,11]. Individuals may succumb to toxic exposure levels [12] or experience sub-lethal effects including diminished reproduction [13], reduced response to threats [14], compromised body condition [15], and suppressed immune function [16]. AR residue concentrations and the number of exposures at which deleterious health effects are observed can vary [17], necessitating further investigation to understand species-specific thresholds of AR toxicity. Exposure to ARs has been documented to increase vulnerability to additional environmental stressors including parasites and pathogens (e.g., notoedric mange in bobcats [*Lynx rufus*] and mountain lions [*Puma concolor*] [16,18]). High rates of exposure and health effects in non-target wildlife have become widespread and widely recognized [19], though the management of exposure poses a major challenge.

Regulation of AR compounds varies greatly and is often lacking [19], but concerns over unintended consequences of AR use have increased given heightened documentation and awareness of the negative impacts to human health and non-target wildlife [20]. Since their earliest uses in the 1940’s, commonly used AR compounds have changed over time [21]. First-generation anticoagulant rodenticides (FGARs) including warfarin, diphacinone, chlorophacinone, and dicoumarol require multiple doses to become lethal and are largely available for public and professional use [20]. The more acutely toxic second-generation anticoagulant rodenticides (SGARs), which are toxic given a single dose, include bromadiolone, brodifacoum, and difethialone. These were developed to address rodent resistance to FGARs [22] and in the US have been largely regulated for application by professionals [20]. Specifically, in 2008 the US Environmental Protection Agency (EPA) modified where and how SGARs were sold (Federal Register E8-12493) in an effort to reduce risks to non-target wildlife. In 2024, they released a final biological evaluation on the effects of ARs on endangered species (Docket ID EPA-HQ-OPP-2023–0567) and suggested mitigation measures for use in critical habitats. Yet, information on the effectiveness of policies in reducing wildlife AR exposure is limited. Most states follow EPA standards for AR use, though some have enacted additional restrictions. Some of the strictest US regulations were enacted in California, which currently bans the use of both FGARs and SGARs outside of agricultural and public health applications (Poison-Free Wildlife Act, AB 2552, 2024). However, exposure in non-target wildlife continues in the state, likely due to purchase of compounds outside registered brokers and illegal application [23]. Trends in carnivore AR exposure remain similar across the World despite variation in regulation [19] and attempts to limit contamination of wildlife and their habitats [24].

In the northeastern US, AR exposure in non-target wildlife, especially mesopredators, is common. Fisher (*Pekania pennanti*) are widespread across the region and experience frequent exposure with multiple AR compounds, with notably high exposure in Vermont and New Hampshire [25,26]. High AR prevalences have also been observed in birds of prey in Massachusetts [27,28], red-tailed hawks (*Buteo jamaicensis*) in New York [29], and mesocarnivores in Pennsylvania [30]. The wildland-urban interface, or interspersing of structures within undeveloped vegetated spaces, appears to be a major driver of AR exposure [25]. Across the northeastern states a greater proportion of the fisher population detected with ARs in their system was correlated with suppression of population growth potential [11]. Notably, rates of exposure in New York fisher increased each year over the course of a three-year study [31]. Regulation of ARs in northeastern states follows federal rules and EPA guidance, though additional regulations banning the sale and distribution of both FGARs and SGARs and prohibiting use around wildlife habitats are currently being considered (New York Senate Bill S9069, Assembly Bill A10012; Vermont H.326; New Hampshire House Bill 1676). Both baseline and longitudinal data are required to evaluate whether regulatory changes produce tangible benefits to wildlife health through reduced rates of exposure or concentrations and how long it takes to see the effects of regulation change. Meanwhile, AR exposure may also change as continued development across the region increases habitat fragmentation [32], expanding the diffusion of rodenticide compounds in wild spaces with the intermixing of human structures and wildlife habitat [11].

Bobcat populations across the Northeast generally appear stable or increasing after rebounding from widespread declines [33,34], although some populations remain small or at low-densities [35,36]. Declines in habitat connectivity are expected to negatively impact bobcats in the Northeast, where much of their habitat is on privately-owned land [37] or has experienced pronounced human modification [38]. Both habitat fragmentation and urban development remain areas of concern for bobcat population health [39,40], while increased incursion of human-dominated landscapes into bobcat habitat could increase exposure to ARs [41] and contribute to population decline [10]. Bobcats are a wide-ranging species frequently monitored by state agencies and sensitive to disturbance. The goal of this study was to leverage long-term data to establish a baseline for bobcat exposure to ARs across a large extent of the Northeast US, with specific objectives to (1) quantify anticoagulant rodenticide contamination in samples from New Hampshire, Vermont, New York, and New Jersey, (2) evaluate changes in AR detection in bobcats across the span of a decade and between different sources of biological samples, and (3) identify spatial hot spots of AR exposure. Based on previous research with fisher in the region [20], and consistent with studies of AR exposure [24], we expected to detect frequent exposures of multiple AR compounds and, further, to observe spatial hot spots of exposure in southern Vermont and New Hampshire. Bobcats are obligate carnivores and their reliance on small mammals as a food source may place them at greater risk of secondary poisoning from ARs compared to other mesocarnivores like fisher, red fox (*Vulpes vulpes*), and grey fox (*Urocyon cinereoargenteus*). However, bobcats appear less tolerant of human activities, avoiding urban and developed lands [42,43] and recreationists [44], although urban bobcats populations have been observed [10] and tend to have higher concentrations of AR residues [16]. Nevertheless, sensitivity to human disturbance [45] potentially reduces the likelihood of bobcats scavenging rodents exposed to ARs compared to fisher [30]. Indeed, in stable isotope samples collected across the Adirondack Park (NY), bobcats exhibited less use of human food sources than coyotes (*Canis latrans*), gray foxes, red foxes [46], and fisher [47]. Thus, although we expected broad-scale patterns in AR exposure to match those observed for fisher across the region, we expected a lower overall magnitude of exposure in bobcats.

## Materials and Methods

### Sample collection and laboratory analyses

Samples were opportunistically obtained from regulated harvest, incidental harvest, and road-killed bobcats in New Hampshire, Vermont, New York, and New Jersey from 2013–2024. Incidental harvest included those specimens obtained as non-target capture in harvest. Legal trapping and hunting seasons occurred in Vermont and New York during the study period while hunting and trapping for bobcats were closed in New Hampshire and New Jersey. Therefore, samples in New Jersey were largely obtained from roadkill and incidental harvest from counties where bobcats are found in the state. In Vermont, samples were collected from either roadkill or carcasses that trappers and hunters were required to submit to the Vermont Fish & Wildlife Department. On rare occasions, samples in New Hampshire and New York were obtained from sick, injured or human-bobcat conflict scenarios where bobcats were euthanized by state biologists. Additionally, dead bobcats were occasionally found opportunistically, with post-mortem necropsy attributing the ultimate cause of mortality to sources such as predation or pathogen infection. These specimens, along with conflict and sick animals, were included in a category of ‘other’ sources. The township location, year of sampling, and cause of death were recorded for each individual. Carcasses were submitted for necropsy at the New Hampshire Veterinary Diagnostic Laboratory (Durham, New Hampshire, USA), New Jersey Department of Environmental Protection Office of Fish and Wildlife Health and Forensics (Hampton, New Jersey, USA), or New York State Department of Environmental Conservation Wildlife Health Unit (Delmar, New York, USA). When no discernable cause of death was identified during necropsy, the source was categorized as unknown.

During necropsy, sex was observed and recorded when genital tracts were present. After necropsy, age was determined to the closest year from tooth cementum annuli of a canine [48] submitted to Matson’s Laboratory (Manhattan, MT, USA). Age was then summarized as either juvenile, including kittens <1 year old and juveniles 1−2 years old, or adult, ≥ 2 years old. We collected 20−100 g of liver tissue from each individual, stored samples at −20 C until analysis, and submitted samples for testing at the New Bolton Center of the Pennsylvania Animal Diagnostic Laboratory System. Samples were screened for 11 ARs using mass spectrometry and processed following the protocol in Facka et al. [30]. Samples were analyzed using the AB SCIEX API 4000 LC-MS/MS (SCIEX, Framingham, Massachusetts, USA) or the Agilent 6470 LC-MS/MS (Agilent Technologies, Santa Clara, California, USA).

We recorded the number of different AR compounds detected in each sample and the concentration of each in mg/kg when levels met or exceeded the method detection limit (MDL). Samples with positive detections below the MDL were considered ‘trace’. Testing equipment changed over time, resulting in a change in MDL for several compounds. For those compounds whose MDL changed, we used the higher MDL value for samples in all years to conservatively categorize trace cases. The detection limit values used were: 0.100 ppm for warfarin, coumafuryl, chlorophacinone, coumachlor, dicoumarol, and bromadiolone, 0.05 ppm for diphacinone and difethialone, 0.025 ppm for brodifacoum and difenacoum, and 0.200 ppm for pindone.

### Patterns and hot spots in AR detection

We considered individuals to be exposed when either a detectable concentration or trace level of a given compound was observed. We then summarized the mean and total concentrations of quantifiable levels of each AR and the total number of AR compounds detected in each individual. To describe patterns of detection in our data, we compared the proportion of cases exposed, the number of ARs, and the mean and total AR concentrations by sex, age, state and sample source using Welch’s t-tests [49] and Chi-squared tests [50]. Due to the large number of comparisons, we used a higher p-value threshold of 0.01 for inferring statistical significance. We examined whether exposure rates varied with bobcat age and year of the study using logistic regression, with a binary response variable of 1 indicating exposure and 0 indicating failure to detect an AR compound. We repeated the test for exposure by year of the study with samples from New Jersey alone, where samples were available across all study years, to assess whether the former test was influenced by the pooling of samples from all states and years. We used an additional linear model to assess the relationship between the number of detected AR compounds and bobcat age.

We investigated spatial clustering of AR exposure using Getis-Ord Gi* statistics. This approach evaluates the clustering of spatial data and estimates the significance of statistically high (hot) or low (cold) local cluster values relative to mean levels [51]. Specific sample coordinates were only available for samples from New Jersey. For the remaining states cases were reported by township. For these samples we generated a random location within a given township to represent each individual bobcat sample. This allowed us to represent each case singly with a unique location and binary exposure status (1 exposed, 0 unexposed), rather than pool data for multiple individuals at the township scale. Analysis at the level of township polygons would either involve information loss if township binary exposure status were considered, or would be heavily influenced by sampling and the total number of cases available if the response variable was the number of exposed cases or proportion exposed.

We first conducted a General G test to assess the global clustering of high or low values before statistically testing for hot and cold spots. We used k = 8 nearest neighbors to create adequate cluster size, as k = 4 neighbors proved too small to generate clusters and k = 16 neighbors encompassed a large portion of all samples within single clusters. The global test used a binary weighting of 1 for neighbors and 0 for other points.

All spatial calculations and visualizations were conducted in program R [52] with packages sf [53], spdep [54], and ggspatial [55]. We defined spatial neighbors with the st_knn function and calculated spatial weights of those neighbors with the st_weights function. Both neighbors and weights were used to calculate the Gi statistic for each point (function local_g_perm) over 999 conditional permutations with the two-sided hypothesis that local clusters could be hot or cold areas of AR exposure. We characterized the significance of points at alpha levels of 0.1 (90% confidence), 0.05 (95% confidence), and 0.01 (99% confidence) based on permutation p-values, where significant positive Gi values were hot spots and significant negative Gi values were cold spots. We mapped all aforementioned levels of significance and considered locations to be hot or cold if they exceeded 90% confidence. Maps were created using U.S. Census Bureau 2022 TIGER/Line shapefiles and package tigris [56].

## Results

We obtained samples from 352 individual bobcats, including 31 from New Hampshire, 188 from New Jersey, 60 from New York, and 73 from Vermont (Table 1). These included 190 roadkill cases, 75 harvested during a regulated season, 67 incidentally harvested, and 20 other (n = 6) or unknown sources (n = 14). Individuals sampled included 169 males (64 juveniles, 60 adults, 45 unknown age), 161 females (53 juveniles, 56 adults, 52 unknown age), and 22 bobcats of unknown sex and age. Samples were obtained in New Hampshire from 2019–2021, in New Jersey from 2013–2024, in New York from 2017–2021, and in Vermont from 2021–2024.

**Table 1:** Summary source, age, and sex of 352 bobcat (*Lynx rufus*) samples from New Hampshire (NH), New Jersey (NJ), New York (NY), and Vermont (VT), USA tested for anticoagulant rodenticide exposure. Age is classified as juvenile (< 2 yo) or adult (≥ 2 yo).

Source	NH	NJ	NY	VT	Total
**Roadkill**					
Adult male	7	13	0	1	**21**
Adult female	6	19	0	3	**28**
Juvenile male	5	33	0	1	**39**
Juvenile female	4	24	0	3	**31**
Age unknown, male	1	15	6	1	**23**
Age unknown, female	0	22	16	0	**38**
Unknown sex or age	0	0	10	0	**10**
Source total	23	126	32	9	**190**
					
**Regulated harvest**					
Adult male	0	0	0	17	**17**
Adult female	0	0	0	16	**16**
Juvenile male	0	0	0	13	**13**
Juvenile female	0	0	0	14	**14**
Age unknown, male	0	0	5	1	**6**
Age unknown, female	0	0	8	0	**8**
Unknown sex or age	0	0	1	0	**1**
Source total	0	0	14	61	**75**
					
**Incidental harvest**					
Adult male	1	21	0	0	**22**
Adult female	4	7	0	0	**11**
Juvenile male	1	8	0	0	**9**
Juvenile female	1	5	0	0	**6**
Age unknown, male	0	14	0	0	**14**
Age unknown, female	0	4	1	0	**5**
Unknown sex or age	0	0	0	0	**0**
Source total	7	59	1	0	**67**
					
**Other/unknown**					
Adult male	0	0	0	0	**0**
Adult female	0	0	0	1	**1**
Juvenile male	1	2	0	0	**3**
Juvenile female	0	0	0	2	**2**
Age unknown, male	0	1	1	0	**2**
Age unknown, female	0	0	1	0	**1**
Unknown sex or age	0	0	11	0	**11**
Source total	1	3	13	3	**20**
					
**Total**	**31**	**188**	**60**	**73**	**352**

Seven of the 11 tested AR compounds were detected: first-generation warfarin, diphacinone, chlorophacinone, and dicoumarol, and second-generation brodifacoum, bromadiolone, and difethialone. Over half of all bobcats sampled (58.5%, n = 206) were exposed at detectable (trace or quantifiable) levels to at least one AR compound, ranging from 40–93.5% by state (Table 2; Fig 1). New Hampshire had the highest proportion of positive cases (93.5%), followed by Vermont (69.9%), New Jersey (54.3%), and New York (40%). In 2021, the only year with overlapping sampling in all states, the proportion of samples with AR detections was 90% in New Hampshire (17 of 19 samples), 73% in Vermont (11 of 15 samples), 67% in New York (4 of 6 samples), and 65% in New Jersey (17 of 26 samples).

**Table 2:** Number of bobcat (*Lynx rufus*) samples collected 2013-2024 with positive detections of ≥1 of the 11 tested anticoagulant rodenticide compounds, separated into detections of trace levels versus quantifiable (> method detection limit) detections only. Percent is the percentage of positive tests from each state.

State	Total tests	Including traces		Quantifiable only	
		Positive	Percent	Positive	Percent
**New Hampshire**	31	29	93.5%	19	61.3%
**New Jersey**	188	102	54.3%	41	21.8%
**New York**	60	24	40.0%	4	6.7%
**Vermont**	73	51	69.9%	16	21.9%
**Total**	352	206	58.5%	80	22.7%

**Fig 1:**
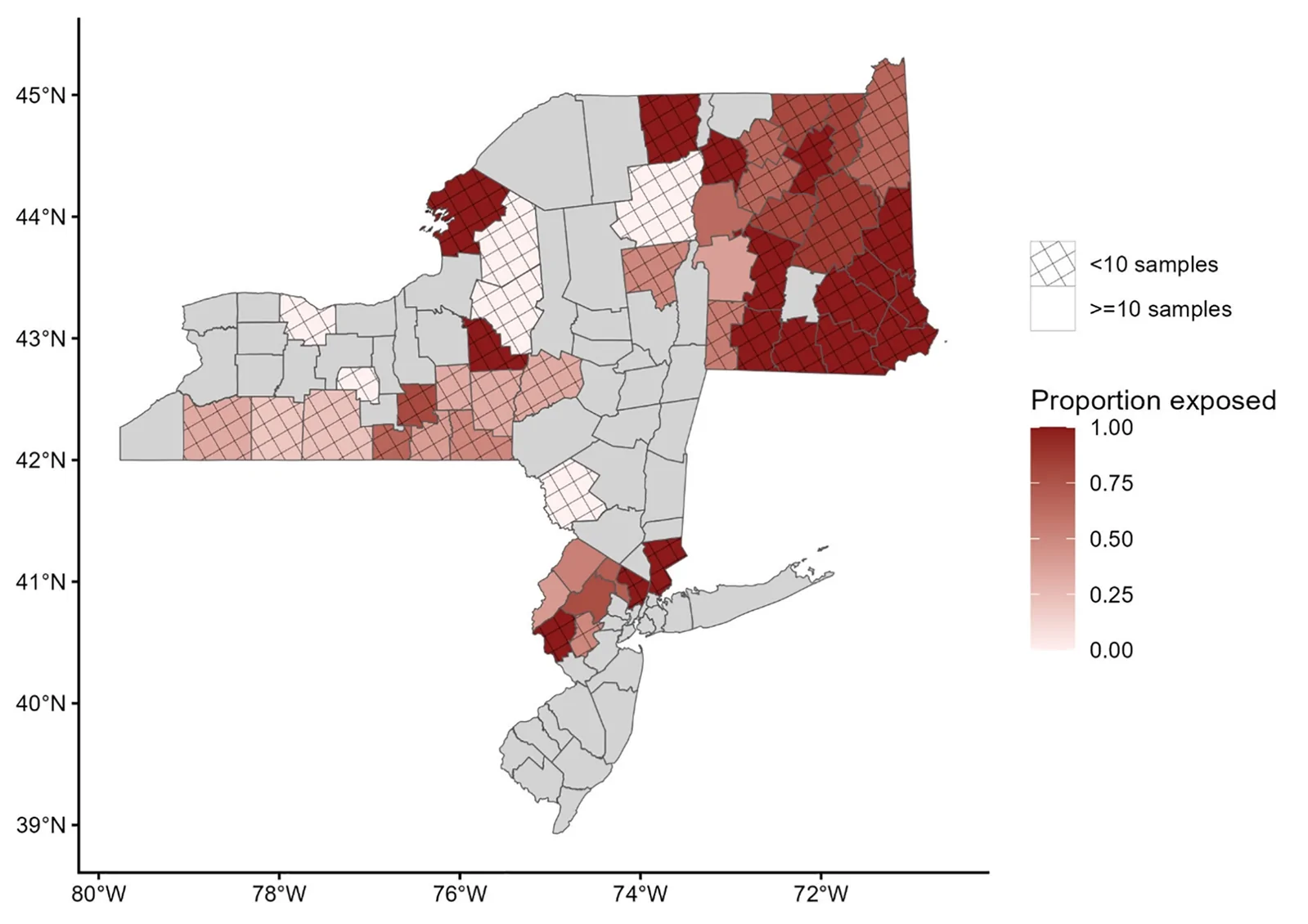
Proportion of bobcat (*Lynx rufus*) samples exposed to ≥1 anticoagulant rodenticide compound. Detections were at trace or quantifiable levels from samples collected in New Jersey, New York, Vermont, and New Hampshire, USA, 2013-2024. Grey polygons are counties with no samples, and crosshatch marks designate counties with fewer than ten samples.

Of the bobcats exposed, we detected between 1 and 4 AR compounds per individual (mean = 1.07, standard deviation [sd]=1.17): 50% (n = 103) were exposed to one AR, 25% (n = 51) were exposed to two ARs, 17% (n = 36) to three ARs, and 8% (n = 16) to four ARs. New Hampshire had the highest mean number of AR compounds (2.19, sd = 1.17), followed by Vermont (mean = 1.22, sd = 1.04), then New Jersey (mean = 1.03, sd = 1.22) and New York (mean = 0.43, sd = 0.56). The majority of exposures were at trace levels (72%, 272 of 377 total detections; Table 3), with the highest number of exposures to diphacinone (n = 116), bromadiolone (n = 114), and brodifacoum (n = 100), and fewer detections of difethialone (n = 38), chlorophacinone (n = 5), dicoumarcol (n = 2), and warfarin (n = 2). Of total compound detections, SGARs (n = 252) were detected more frequently than FGARs (n = 125). More individual bobcats had exposures to SGARs (n = 159, 45%) than FGARs (n = 119, 34%), with approximately 20% being exposed to both. For quantifiable levels of detection, mean concentrations were low (Table 4), with the highest concentrations observed for bromadiolone (mean = 0.57 ppm, sd = 0.73), followed by dicoumarol (0.21 ppm), difethialone (mean = 0.17 ppm, sd = 0.17), diphacinone (mean = 0.17 ppm, sd = 0.14), and brodifacoum (mean = 0.12 ppm, sd = 0.14). Using MDL values unique to each time period, summarized concentrations were slightly lower for bromadiolone (mean = 0.42 ppm, sd = 0.66) and brodifacoum (mean = 0.11 ppm, sd = 0.13).

**Table 3:** Detections of first and second-generation anticoagulant rodenticide compounds in bobcat (*Lynx rufus*) across northeastern US states (NH = New Hampshire, NJ = New Jersey, NY = New York, VT = Vermont) sampled 2013-2024, separated into those at trace levels versus quantifiable levels (> method detection limit).

	NH	NJ	NY	VT	Total
**First generation**					
**Diphacinone**					
Quantifiable	14	5	3	4	26
Trace	13	43	15	19	90
All	27	48	18	23	116
**Chlorophacinone**					
Quantifiable	0	0	0	0	0
Trace	4	1	0	0	5
All	4	1	0	0	5
**Dicoumarol**					
Quantifiable	0	0	1	0	1
Trace	0	0	0	1	1
All	0	0	1	1	2
**Warfarin**					
Quantifiable	0	0	0	0	0
Trace	0	0	0	2	2
All	0	0	0	2	2
**Second generation**					
**Bromadiolone**					
Quantifiable	6	28	0	6	40
Trace	10	40	4	20	74
All	16	68	4	26	114
**Brodifacoum**					
Quantifiable	13	13	0	10	36
Trace	7	21	2	23	64
All	20	44	3	33	100
**Difethialone**					
Quantifiable	0	2	0	0	2
Trace	1	31	0	4	36
All	1	33	0	4	38

**Table 4:** Mean concentrations of quantifiable levels of anticoagulant rodenticides detected in bobcats (*Lynx rufus*) sampled 2013-2024 in New Hampshire (NH), New Jersey (NJ), New York (NY), and Vermont (VT), USA. Standard deviations (sd) are provided parenthetically when sample sizes (n) were >1.

State	Diphacinone		Dicoumarol		Brodifacoum		Bromadiolone		Difethialone	
	x̄ (sd)	n	x̄ (sd)	n	x̄ (sd)	n	x̄ (sd)	n	x̄ (sd)	n
**NH**	0.19 (0.13)	14	–	–	0.13 (0.11)	13	0.31 (0.14)	6	–	–
**NJ**	0.07 (0.04)	5	–	–	0.08 (0.06)	13	0.70 (0.84)	28	0.17 (0.17)	2
**NY**	0.22 (0.17)	3	0.21	1	–	–	–	–	–	–
**VT**	0.17 (0.18)	4	–	–	0.18 (0.21)	10	0.23 (0.18)	6	–	–
**Overall**	0.17 (0.14)	26	0.21	1	0.12 (0.14)	36	0.57 (0.73)	40	0.17 (0.17)	2

In testing for significant differences in exposure between groups, we found no difference between males and females in exposure rates (t = 1.60, p = 0.11), number of AR compounds (t = 1.44, p = 0.15), mean concentration (t = −0.17, p = 0.86), or total concentration (t = 0.70, p = 0.48). We did, however, detect differences by bobcat age in the number of AR compounds (χ^2^ = 77.54, p < 0.01) and total AR concentration (χ^2^ = 1113.7, p < 0.001; Fig 2). The odds of AR exposure increased by approximately 35% with each annual increase in age (odds ratio (OR) 1.35, 95% confidence interval (CI) [1.16, 1.60]), whereas the mean number of AR compounds they were exposed to increased by 0.17 per year of age (adjusted R^2^ = 0.09, p < 0.001). Mean concentration did not differ by bobcat age (χ^2^ = 577.1, p = 0.34). The number of AR compounds per individual (χ^2^ = 28.3, p = 0.01) and the proportion exposed (χ^2^ = 12.04, p = 0.01) both differed by sample source, although total concentration (χ^2^ = 168.2, p = 0.67) and mean concentration (χ^2^ = 190.9, p = 0.23) did not. We detected ARs in samples from incidental and regulated harvest more often and in higher quantities than in the road kill and other sources. States had significantly different proportions of samples exposed (χ^2^ = 29.43, p < 0.01), number of AR compounds detected (χ^2^ = 78.83, p < 0.01), and total concentration per sample (χ^2^ = 250.86, p < 0.01), though mean concentration did not differ (χ^2^ = 165.09, p = 0.72). Specifically, New York had a smaller proportion exposed, while Vermont and New Hampshire had higher rates of exposure. New Hampshire had more samples with three ARs than expected and fewer with zero ARs, while New York had fewer cases with higher numbers of AR compounds. Finally, combining all samples revealed a positive relationship between AR exposure and year of sampling for both exposure to any AR compound (OR 1.25, 95% CI [1.15, 1.36]) and exposure to SGARs (OR 1.37, 95% CI [1.24, 1.51]) specifically (Fig 3). Exposure to FGARs had a weaker relationship with study year (OR 1.06, 95% CI [0.98, 1.16]). The odds of exposure in the sample increased by approximately 6% per year for FGARs and 37% per year for SGARs, or 25% for any generation of compound. We repeated this test for samples only from New Jersey, the only state sampled in all years of the study, and found a similar pattern of increasing odds of exposure to either generation at a rate of 25% per year (OR 1.25, 95% CI [1.13, 1.40]). This included a significant relationship for both FGARs (OR 1.16, 95% CI [1.03 to 1.33]) and SGARs (OR 1.32, 95% CI [1.18, 1.50]).

**Fig 2:**
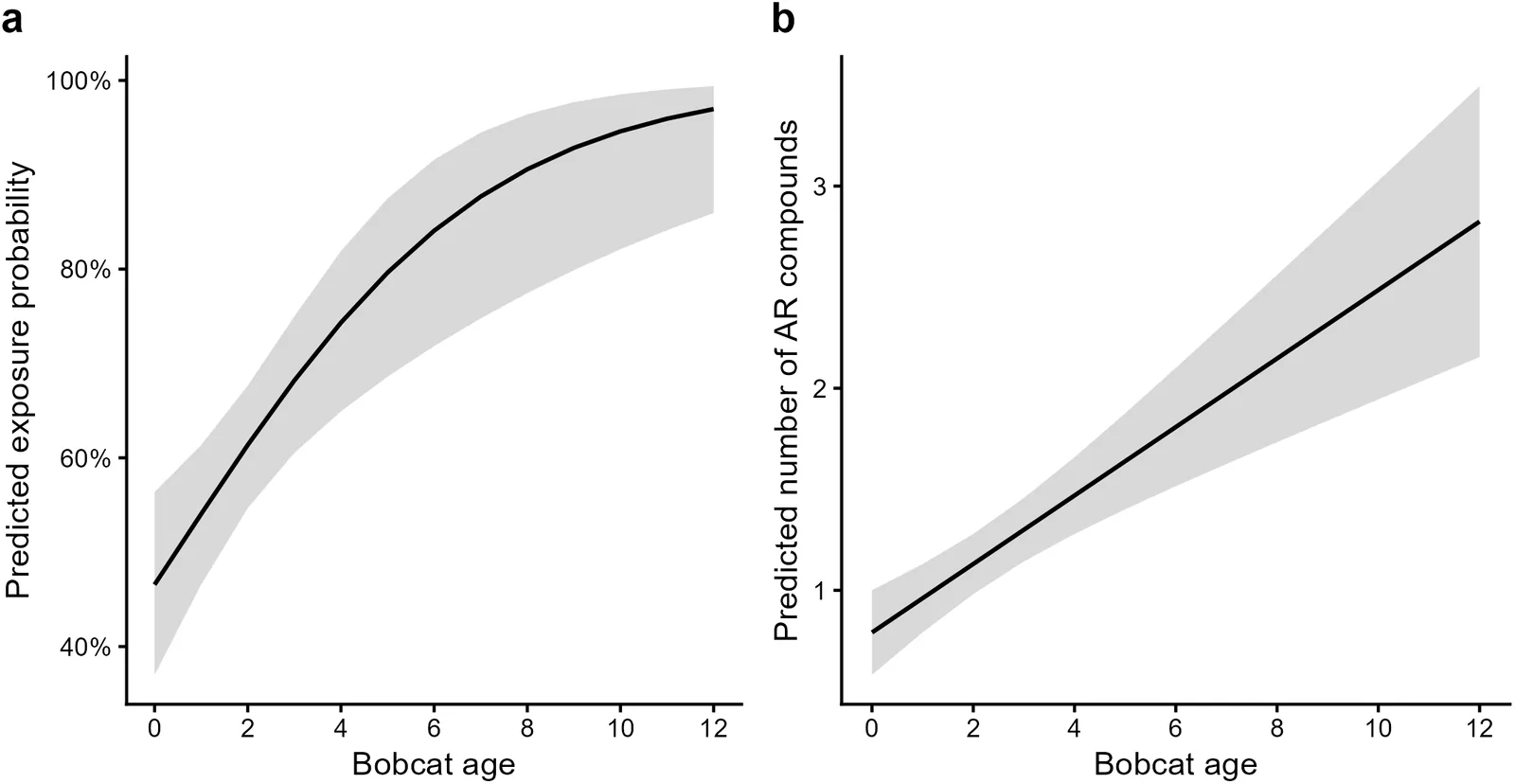
Relationship between bobcat (*Lynx rufus*) age and anticoagulant rodenticide detection. Anticoagulant rodenticide (AR) exposure rates (a) and the number of AR compounds (b) were predicted to increase with increasing bobcat age. Lines are regressions and shaded regions 95% confidence intervals.

**Fig 3:**
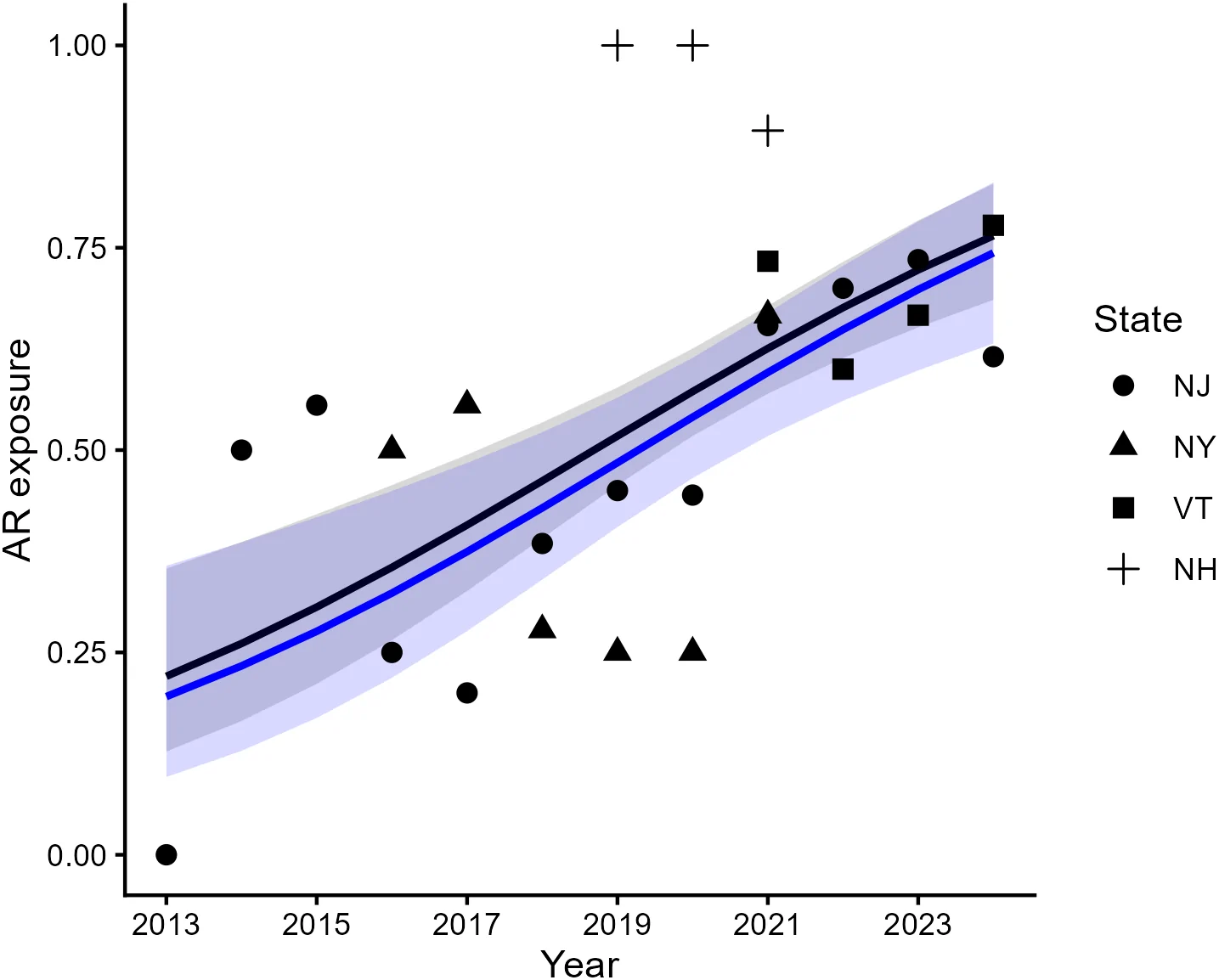
Predicted bobcat (*Lynx rufus*) exposure to ≥1 anticoagulant rodenticide compound by study year. Presented are the logistic regression predictions (lines) and 95% confidence intervals (shaded region) for detection of any of the 11 tested anticoagulant rodenticide (AR) compounds in all samples combined (black) and for New Jersey samples alone (blue). Points are the observed proportion of samples exposed by state in a given year (circles for New Jersey [NJ], triangles for New York [NY], squares for Vermont [VT] and pluses for New Hampshire [NH]).

For the analysis of hot spots of AR exposure (Fig 4), township data was available for 29 samples from New Hampshire, 48 samples from New York, and all 73 samples from Vermont; point location data were available for all 188 samples from New Jersey. Of the 12 samples not included from New York, three were exposed to ARs; of the two samples not included from New Hampshire, one was exposed. The Getis-Ord global G test demonstrated that spatial clusters of AR exposure existed in the dataset (Global G = 0.026, Expected G = 0.024, z = 3.76, p < 0.01).

**Fig 4:**
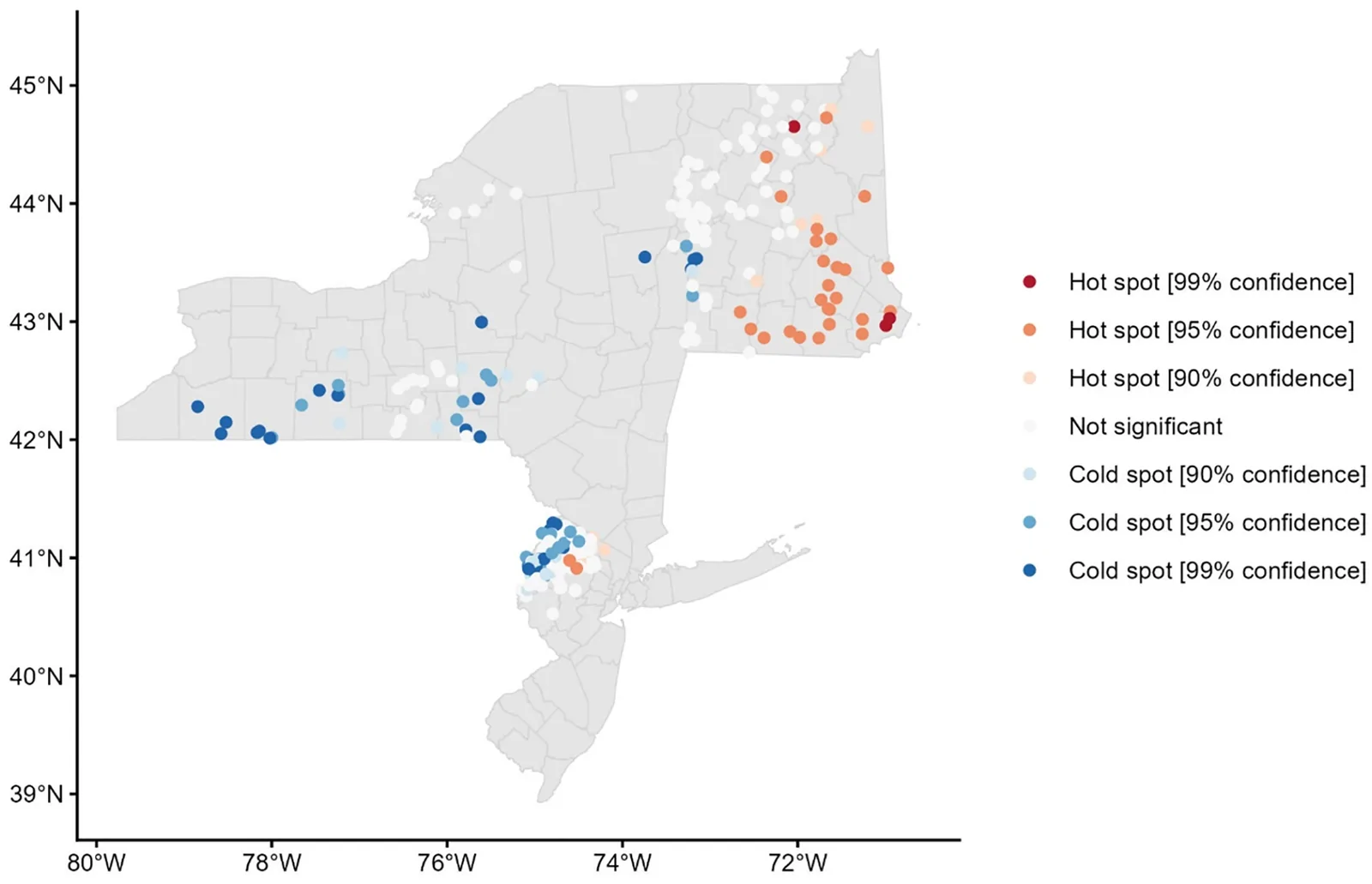
Hot and cold spots of anticoagulant rodenticide exposure in northeastern bobcat (*Lynx rufus*). Samples were collected from New Hampshire, Vermont, New York, and New Jersey, USA, 2013-2024. Brackets provide statistical significance for Getis-Ord Gi* hot spot assignments at alpha levels of 0.01 (99% confidence), 0.05 (95% confidence), and 0.1 (90% confidence).

Gi statistics ranged from −3.47 to 2.37, which, when paired with the folded permutation p-values, resulted in the classification of 45 hot spots (13%), 107 cold spots (32%), and 186 points lacking statistical significance (55%). All points of statistical significance (>90%) in New York were cold spots (n = 27 [6 at 90%, 8 at 95%, 13 at 99% confidence], 56%) whereas all significant spots in New Hampshire were hot spots (n = 26 [4 at 90%, 20 at 95%, 2 at 99% confidence], 90%). Vermont had 1.5 times as many points categorized as hot spots (n = 9 [3 at 90%, 5 at 95%, 1 at 99% confidence], 12%) compared to cold spots (n = 6 [1 at 90% confidence, 2 at 95%, 3 at 99% confidence], 8%), and New Jersey had over seven times more cold spots (n = 74 [21 at 90%, 26 at 95%, 27 at 99% confidence], 39%) than hot spots (n = 10 [8 at 90%, 2 at 95% confidence], 5%). Hot spots with the highest confidence were concentrated in the southeastern portion of New Hampshire along with eastern Vermont and northcentral New Jersey.

## Discussion

Our study detected high rates of AR exposure and steadily increasing exposure over time in bobcats sampled across four northeastern US states. The majority of bobcats in our sample (58.5%) were exposed to at least one AR, consistent with but generally at lower magnitudes than observed for fisher (79% [25]) and raptors (100% [27]) in the northeastern U.S., bobcats (89% [14]) and coyotes (98% [57]) in California, and higher than mesocarnivores in Pennsylvania (44% [30]). We detected high rates of brodifacoum and bromadiolone exposure, with more detections of SGARs than FGARs. However, our high detection rates of the FGAR diphacinone echo regional differences of high detections in the Northeast [25] and in urban coyotes in California [57], but not fisher and bobcat in California [14,58]. Such continued exposure to FGARs could indicate continued use of the compound [59], persistence on the landscape, or increased use of FGARs as regulations for the more potent SGARs become increasingly strict. We did not detect the FGAR pindone or SGAR difenacoum, reflecting limited use in the US relative to other countries [60,61]. Exposure to multiple AR compounds in bobcats was also consistent with the aforementioned northeastern US studies, with half of our positive samples exposed to one AR and the other half indicating multiple AR exposures. Quantifiable concentrations of ARs spanned the range associated with mortality from notoedric mange [18], although threshold levels for direct mortality and toxicosis are lacking [14]. Other studies of bobcats have detected small numbers of direct mortality from AR toxicosis [18], as well as gastrointestinal bleeding [62], population-level impacts [10], and increased susceptibility to environmental stressors [16] from AR exposure. Although bobcat populations are generally increasing across their range in North America [33], the threat of ARs to both individual and local population health should continue to be monitored as an important consideration for wildlife management.

We detected hot spots of rodenticide exposure across multiple northeastern states, primarily in New Hampshire and portions of Vermont and New Jersey. This spatial pattern and the proportion of sample exposed is broadly similar to exposure status and the number of compounds detected in fisher across the Northeast [25], although some differences exist because we lacked samples across southern New York and did not interpolate across a continuous spatial surface. Specifically, fisher exposure to ARs was high in southern and western New York, though we did not detect any hot spots for bobcat in the state. We note that Getis-Ord Gi* may be sensitive to unequal sampling and have reduced confidence in areas without adequate neighbors [63]. The statistic also performs less well than other metrics of spatial autocorrelation when low-value samples are clustered [64]. Because of these limitations, cold spots in states with relatively sparse sampling (i.e., New York) may underrepresent local hot spots. Additional sampling may help elucidate whether New York is truly as cold, and New Hampshire truly as hot, as they appear given our available samples. Random chance and underlying patterns in sampling or biological variation may also have influenced patterns in states where we had limited sample sizes and temporal variation in sampling. In contrast, extensive sampling in New Jersey revealed a mix of hot and cold spots. Fisher are relatively rare in New Jersey [65], potentially making bobcat a better sentinel species for AR exposure across the southern edge of fisher range. Broad-scale patterns of exposure for bobcats may be similar to fishers if driven by the intermixing of low-density structures with forested wildland [25]; fine-scale patterns may differ from fisher due to increased bobcat use of edge habitats [37] and avoidance of human disturbance [46]. Edge habitat could be particularly important for AR exposure, considering the interface of housing units and altered open spaces experiences high residential AR use [66]; however, exposure in remote natural areas also occurs [14]. The urban-wildland edge was also found to be important for bobcat physiological responses to ARs at fine spatial scales in California [16]. Yet, studies frequently report limited or no association between habitat types and exposure [19,67], reflecting the ubiquity of ARs across the landscape.

Despite increasing awareness of the negative effects of ARs on non-target wildlife and federal restrictions on consumer access to SGARs, exposure to widespread taxa persists and, as we observed, has been steadily increasing over time. EPA modifications of consumer access to SGARs by the EPA in 2008 may not have stalled overall AR exposure risk, though specific compound availability may have changed [7]. For instance, multiple species of owls were exposed to increasing concentrations of bromadiolone yet decreasing concentrations of brodifacoum over time [68], while red-tailed hawks saw increasing frequencies of bromadiolone and difethialone detections despite regulation change going fully into effect in 2015 [27]. Sales of bromadiolone, the compound detected at the highest mean concentration in our study, were reported to have increased during the study period [68]. Our detected rates of increase were similar to 8% annual increases in exposure of fisher in New York [31]. We detected a higher rate of increase for SGARs than FGARs despite higher perceived risk and tighter restrictions on their sale and use [69]. SGARs are often preferred by applicators because of their higher single-dose potency and lower resistance by rodents. While demand for both generations of rodent control products is expected to continue to increase, growth of the FGAR market may surpass that of SGARs [59] considering potential future restrictions or bans of SGARs. Still, both FGARs and SGARs remain commonly available online, and misuse by the public contributes to exposure even after regulation [70]. Overall, studies that monitor changes in percentage of samples positive for ARs indicate increases in overall exposure over multiple decades [68,71], with variation in when certain compounds were detected most [14,27]. Increasing fragmentation and development of the Northeast [32], along with greater intrusion of toxicants into wild spaces, could further increase exposure rates and contamination risk to non-target species [67]. Importantly, temporal variation in our sample was partially conflated with spatial variation in sample collection; samples from New Hampshire had higher rates of exposure and were collected in later years. However, the trend of increasing concentrations over time was consistent in New Jersey, where samples were obtained across all years. Though the collective sample also points to an increase over time, regional studies that consistently monitor AR exposure would be helpful in assessing trends in relation to legislation changes.

Both the mean number of compounds and the proportion of bobcats exposed in our study increased with bobcat age, consistent with patterns observed in mesocarnivores in Chicago [72] and coyotes in California [57]. Although ARs are metabolized in animal tissue- typically faster for FGARs than SGARs- concentrations may accumulate if intake outpaces excretion rates [9] and potentially accumulate over an individual’s lifetime. Still, individuals <1 year old in our sample experienced high rates of exposure, with almost 50% of sampled kittens exposed. This may result from both the consumption of small contaminated prey items, including invertebrates [73], and maternal transfer [14,72], reflecting not only ubiquitous exposure across the landscape but persistent risk over time. Male bobcats were expected to have higher AR concentrations and exposure rates due to greater dispersal and larger home ranges than females [74], but we did not detect differences in exposure rates by sex. Differences in exposure were observed across age and sex for fisher in NY [31], but not detected in bobcats [16] or coyotes [57] sampled in California. We report concentrations of AR residues observed within our sample, but primarily analyzed rates of exposure and number of AR compounds, considering concentration does not necessarily reflect time since consumption or probability of negative health consequences [7].

Sampling for this study was largely opportunistic and varied by state, which could potentially have affected the variation in AR exposure rates observed among states and skewed our sample compared to the regional bobcat population. The sampling of road-killed animals could over-represent AR-exposed animals if they were predisposed to mortality; however, our samples from incidental and regulated harvest showed higher rates of exposure than roadkill samples. Conversely, bobcats that are overtly sick or die from AR exposure could be missed or misrepresented by opportunistic sampling closer to roads and development [75], potentially leading studies, even with large sample sizes, to fail to estimate true exposure rates or obscure assessments of landscape drivers. Additionally, detections of FGARs in our sample may be underrepresented considering their shorter half-life and lower dose in single exposures [8]. Difficulties in sampling across large regions and quantifying exposure in available samples has necessitated broad-scale work as in this study. As such, it was necessary to pool information across states to analyze changes over time, potentially conflating spatial and temporal differences. Variation in population densities across broad areas also makes obtaining consistent sample sizes challenging. However, biases of convenience sampling may have been comparable across the sampled states, and the patterns observed are largely consistent with measurements of other taxa [25,30]. Because patterns of exposure were similar to fisher in the Northeast [25], bobcat could potentially serve as a sentinel species in northern states where they are common and/or harvested [11]. For states where bobcats and fisher are comparatively rare [33,65], wide-ranging mesocarnivores such as foxes could be monitored as indicators of exposure. Expanded analysis of bobcat samples from adjacent states in New England would help to broaden the scale of inference and inform patterns and drivers of hot spots, particularly those along the southeastern portion of New Hampshire. Additionally, consistent monitoring of collected samples over time would improve our ability to assess temporal trends in addition to spatial variation.

## Conclusion

Anticoagulant rodenticides pose an important yet challenging-to-manage risk to non-target wildlife species through both direct mortality and indirect impacts to individual and population health. Despite continued detection and our increasing knowledge of the deleterious effects of AR exposure, current regulations have failed to mitigate risks to non-target wildlife, as demonstrated by our finding of high and growing exposure in bobcats across the northeastern US. Mitigation measures have not reduced overall exposure to ARs; rather, persistent use for rodent control has led to consistent secondary exposure. While the built environment of the Northeast poses risks of increased incursion of AR compounds into wildlands, public educational campaigns on correct use and rodent carcass disposal, along with restrictions on deployment of SGARs, may help reverse trends of increasing exposure rates. Additional research on the sublethal effects of ARs in bobcats and other wildlife, the additive and interactive effects of ARs with other determinants of wildlife health, and evaluation of educational programs for minimizing wildlife risks are needed.

## Supporting information

S1 File Data used in northeastern bobcat anticoagulant rodenticide exposure analysis. Included are data used in analysis of bobcat anticoagulant exposure across the northeastern US. Provided are the state and year of sampling, sample source, bobcat age and sex, and county location, as well as binary exposure status, the number of anticoagulant rodenticide compounds detected, and concentrations or trace detections by compound.
(CSV)

## References

[1] Kumar S. Environmental contaminants and their impact on wildlife. In: Ahmad MI, Mahamood M, Javed M, Alhewairini SS, editors. Toxicology and human health: Environmental exposures and biomarkers. Singapore: Springer Nature. 2023. p. 3–26. doi: 10.1007/978-981-99-2193-5_1

[2] Mockrin MH, Stewart SI, Radeloff VC, Hammer RB, Johnson KM. Spatial and temporal residential density patterns from 1940 to 2000 in and around the Northern Forest of the Northeastern United States. Popul Environ. 2013;34(3):400–19. doi: 10.1007/s11111-012-0165-5

[3] König HJ, Kiffner C, Kramer-Schadt S, Fürst C, Keuling O, Ford AT. Human–wildlife coexistence in a changing world. Conservation Biology. 2020;34:786–94. doi: 10.1111/cobi.13513 32406977

[4] Fairbrother A. Federal environmental legislation in the U.S. for protection of wildlife and regulation of environmental contaminants. Ecotoxicology. 2009;18(7):784–90. doi: 10.1007/s10646-009-0355-9 19562483

[5] Rands MRW, Adams WM, Bennun L, Butchart SHM, Clements A, Coomes D. Biodiversity Conservation: Challenges Beyond 2010. Science. 2010;329(5997):1298–303. doi: 10.1126/science.1189138 20829476

[6] Keane A, Jones JPG, Edwards-Jones G, Milner-Gulland EJ. The sleeping policeman: understanding issues of enforcement and compliance in conservation. Animal Conservation. 2008;11(2):75–82. doi: 10.1111/j.1469-1795.2008.00170.x

[7] Rattner BA, Lazarus RS, Elliott JE, Shore RF, van den Brink N. Adverse outcome pathway and risks of anticoagulant rodenticides to predatory wildlife. Environ Sci Technol. 2014;48(15):8433–45. doi: 10.1021/es501740n 24968307

[8] Rattner BA, Harvey JJ. Challenges in the interpretation of anticoagulant rodenticide residues and toxicity in predatory and scavenging birds. Pest Manag Sci. 2021;77(2):604–10. doi: 10.1002/ps.6137 33052019

[9] López-Perea JJ, Mateo R. Secondary exposure to anticoagulant rodenticides and effects on predators. In: Van Den Brink NW, Elliott JE, Shore RF, Rattner BA, editors. Anticoagulant rodenticides and wildlife. Cham: Springer International Publishing. 2018. p. 159–93. doi: 10.1007/978-3-319-64377-9_7

[10] Keating MP, Bodinof Jachowski CM, Diefenbach DR, Jordan JD, Nemeth NM, Rainwater TR, et al. Anticoagulant rodenticides contribute to a decline in an Urban Carnivore. Animal Conservation. 0:1–12. doi: 10.1111/acv.70061

[11] Silveira G, Frair JL, Cohen J, Watson M, Tate P, Royar K, et al. Anticoagulant rodenticides may affect fisher population trends in the northeastern United States. J Wildl Manag. 2025;89(4). doi: 10.1002/jwmg.22727

[12] Niedringhaus KD, Nemeth NM, Gibbs S, Zimmerman J, Shender L, Slankard K, et al. Anticoagulant rodenticide exposure and toxicosis in bald eagles (Haliaeetus leucocephalus) and golden eagles (Aquila chrysaetos) in the United States. PLoS One. 2021;16(4):e0246134. doi: 10.1371/journal.pone.0246134 33826627 PMC8026043

[13] Robinson MH, Twigg LE, Wheeler SH, Martin GR. Effect of the anticoagulant, pindone, on the breeding performance and survival of merino sheep, Ovis aries. Comp Biochem Physiol B Biochem Mol Biol. 2005;140(3):465–73. doi: 10.1016/j.cbpc.2004.11.011 15694595

[14] Serieys LEK, Armenta TC, Moriarty JG, Boydston EE, Lyren LM, Poppenga RH, et al. Anticoagulant rodenticides in urban bobcats: Exposure, risk factors and potential effects based on a 16-year study. Ecotoxicology. 2015;24(4):844–62. doi: 10.1007/s10646-015-1429-5 25707484

[15] Elmeros M, Christensen TK, Lassen P. Concentrations of anticoagulant rodenticides in stoats Mustela erminea and weasels Mustela nivalis from Denmark. Sci Total Environ. 2011;409(12):2373–8. doi: 10.1016/j.scitotenv.2011.03.006 21477845

[16] Serieys LEK, Lea AJ, Epeldegui M, Armenta TC, Moriarty J, VandeWoude S. Urbanization and anticoagulant poisons promote immune dysfunction in bobcats. Proc R Soc B Biol Sci. 2018;285(1871):20172533. doi: 10.1098/rspb.2017.2533 PMC5805946 29343604

[17] Elliott JE, Silverthorn V, English SG, Mineau P, Hindmarch S, Thomas PJ, et al. Anticoagulant rodenticide toxicity in terrestrial raptors: Tools to estimate the impact on populations in North America and globally. Environ Toxicol Chem. 2024;43(5):988–98. doi: 10.1002/etc.5829 38415966

[18] Riley SPD, Bromley C, Poppenga RH, Uzal FA, Whited L, Sauvajot RM. Anticoagulant Exposure and Notoedric Mange in Bobcats and Mountain Lions in Urban Southern California. J Wildl Manag. 2007;71(6):1874–84. doi: 10.2193/2005-615

[19] Keating MP, Saldo EA, Frair JL, Cunningham SA, Mateo R, Jachowski DS. Global review of anticoagulant rodenticide exposure in wild mammalian carnivores. Animal Conservation. 2024;27(5):585–99. doi: 10.1111/acv.12947

[20] Eisemann JD, Fisher PM, Buckle A, Humphrys S. An International Perspective on the Regulation of Rodenticides. In: Van Den Brink NW, Elliott JE, Shore RF, Rattner BA, editors. Anticoagulant Rodenticides and Wildlife. Cham: Springer International Publishing. 2018. p. 287–318. doi: 10.1007/978-3-319-64377-9_11

[21] Witmer G, Eisemann JD. Rodenticide use in rodent management in the United States: an overview. https://digitalcommons.usu.edu/wdmconference/2007/session2/7/. 2007. Accessed 2026 March 26.

[22] Hadler MR, Buckle AP. Forty-five years of anticoagulant rodenticides--past, present and future trends. Proc Vertebr Pest Conf. 1992;15(15).

[23] Hofstadter DF, Kryshak NF, Gabriel MW, Wood CM, Wengert GM, Dotters BP, et al. High rates of anticoagulant rodenticide exposure in California Barred Owls are associated with the wildland–urban interface. Ornithological Applications. 2021;123(4):duab036. doi: 10.1093/ornithapp/duab036

[24] Fernández-Casado D, García-Muñoz J, Portillo-Moreno Á, Martínez-Morcillo S, Míguez-Santiyán MP, Pérez-López M. Anticoagulant rodenticides in mesocarnivores around the world: A review. Environ Chem Ecotoxicol. 2025;7:966–79. doi: 10.1016/j.enceco.2025.05.009

[25] Silveira G, Frair JL, Murphy L, Ellis JC, Needle D, Cunningham SA. Drivers of anticoagulant rodenticide exposure in fishers (Pekania pennanti) across the northeastern United States. Front Ecol Evol. 2024;12:1304659.

[26] Buckley JY, Needle DB, Royar K, Cottrell W, Tate P, Whittier C. High prevalence of anticoagulant rodenticide exposure in New England Fishers (Pekania pennanti). Environ Monit Assess. 2023;195(11):1348. doi: 10.1007/s10661-023-11919-x 37857759

[27] Murray M. Continued Anticoagulant Rodenticide Exposure of Red-tailed Hawks (Buteo jamaicensis) in the northeastern United States with an Evaluation of Serum for Biomonitoring. Environ Toxicol Chem. 2020;39(11):2325–35. doi: 10.1002/etc.4853 33405327

[28] Murray M, Cox EC. Active metabolite of the neurotoxic rodenticide bromethalin along with anticoagulant rodenticides detected in birds of prey in the northeastern United States. Environ Pollut. 2023;333:122076. doi: 10.1016/j.envpol.2023.122076 37336352

[29] Hopf-Dennis C, Kaye S, Hollingshead N, Brooks M, Bunting E, Abou-Madi N. Prevalence of anticoagulant rodenticide exposure in red-tailed hawks (Buteo jamaicensis) and utility of clotting time assays to detect coagulopathy. Ecotoxicology. 2022;31(6):919–32. doi: 10.1007/s10646-022-02558-y 35622198

[30] Facka A, Frair J, Keller T, Miller E, Murphy L, Ellis JC. Spatial patterns of anticoagulant rodenticides in three species of medium-sized carnivorans in Pennsylvania. Can J Zool. 2024;102(5):443–54. doi: 10.1139/cjz-2023-0131

[31] Cunningham SA, Frair JL, Jensen PG, Schuler KL. Spatiotemporal drivers of rodenticide exposure in a mammalian forest carnivore. Sci Total Environ. 2025;982:179605. doi: 10.1016/j.scitotenv.2025.179605 40373683

[32] Adams AB, Pontius J, Galford G, Gudex-Cross D. Simulating forest cover change in the northeastern US: decreasing forest area and increasing fragmentation. Landsc Ecol. 2019;34(10):2401–19.

[33] Roberts NM, Crimmins SM. Bobcat Population Status and Management in North America: Evidence of Large-Scale Population Increase. J Fish and Wildlife Manag. 2010;1(2):169–74. doi: 10.3996/122009-jfwm-026

[34] Carroll RP, Litvaitis MK, Foxall T. Bobcat hair cortisol correlates with land use and climate. J Wildl Manag. 2021;85(4):772–81. doi: 10.1002/jwmg.22029

[35] Mayer AE, Jr TJM, Sullivan ME, Brown C, Husband TP, Gerber BD. Population genetics and spatial ecology of bobcats (Lynx rufus) in a landscape with a high density of humans in New England. Northeast Naturalist. 2021;28(4):408–29. doi: 10.1656/045.028.0401

[36] Mayer AE, McGreevy TJ, Brown C, Ganoe LS, Gerber BD. Transient persistence of bobcat (Lynx rufus) occurrence throughout a human‐dominated landscape. Popul Ecol. 2022;64(4):323–35.

[37] Farrell LE, Levy DM, Donovan T, Mickey R, Howard A, Vashon J, et al. Landscape connectivity for bobcat (Lynx rufus) and lynx (Lynx canadensis) in the Northeastern United States. PLoS One. 2018;13(3):e0194243. doi: 10.1371/journal.pone.0194243 29590192 PMC5874025

[38] Cerreta AL, McCarthy KP, Fowles G. Habitat suitability and landscape connectivity for an expanding population of bobcats. Landscape Ecology. 2023;38(6):1571–89. doi: 10.1007/s10980-023-01639-5

[39] Kozakiewicz CP, Burridge CP, Funk WC, Salerno PE, Trumbo DR, Gagne RB, et al. Urbanization reduces genetic connectivity in bobcats (Lynx rufus) at both intra- and interpopulation spatial scales. Mol Ecol. 2019;28(23):5068–85. doi: 10.1111/mec.15274 31613411

[40] Chmura HE, Fowles G, Pilgrim KL, Strand JM, Theobald DM, Zeller KA, et al. The impact of urbanization on genetic connectivity of 10 mammal species in New Jersey. Ecol Appl. 2025;35(7):e70113. doi: 10.1002/eap.70113 41047850

[41] Rodríguez-Estival J, Mateo R. Exposure to anthropogenic chemicals in wild carnivores: a silent conservation threat demanding long-term surveillance. Current Opinion Environ Sci Health. 2019;11:21–5. doi: 10.1016/j.coesh.2019.06.002

[42] RILEY SPD. Spatial Ecology of Bobcats and Gray Foxes in Urban and Rural Zones of a National Park. Journal of Wildlife Management. 2006;70(5):1425–35. doi: 10.2193/0022-541x(2006)70[1425:seobag]2.0.co;2

[43] Ordeñana MA, Crooks KR, Boydston EE, Fisher RN, Lyren LM, Siudyla S. Effects of urbanization on carnivore species distribution and richness. J Mammal. 2010;91(6):1322–31. doi: 10.1644/09-MAMM-A-312.1

[44] George SL, Crooks KR. Recreation and large mammal activity in an urban nature reserve. Biol Conserv. 2006;133(1):107–17. doi: 10.1016/j.biocon.2006.05.024

[45] Hubbard T, Cove MV, Green AM, Iannarilli F, Allen ML, LaRose SH, et al. Human presence drives bobcat interactions among the U.S. carnivore guild. Biodivers Conserv. 2022;31(11):2607–24. doi: 10.1007/s10531-022-02445-2

[46] Warsen SA, Frair JL, Teece MA. Isotopic investigation of niche partitioning among native carnivores and the non-native coyote (Canis latrans). Isotopes Environ Health Stud. 2014;50(3):414–24. doi: 10.1080/10256016.2014.897946 24666214

[47] Warsen SA. Evolving niche of coyotes in the Adirondack mountains of New York: long-term dietary trends and interspecific competition. Syracuse, New York: State University of New York College of Environmental Science and Forestry. 2012.

[48] Hiller TL, Nistler C, Reding D, White PA, Bled F. Sex identification and age estimation of bobcats and implications for management. Wildl Soc Bull. 2022;46(3):e1328. doi: 10.1002/wsb.1328

[49] Ruxton GD. The unequal variance t-test is an underused alternative to Student’s t-test and the Mann–Whitney U test. Behav Ecol. 2006;17(4):688–90. doi: 10.1093/beheco/ark016

[50] McHugh ML. The chi-square test of independence. Biochem Med (Zagreb). 2013;23(2):143–9. doi: 10.11613/bm.2013.018 23894860 PMC3900058

[51] Getis A, Ord JK. The analysis of spatial association by use of distance statistics. Geogr Anal. 1992;24(3):189–206. doi: 10.1111/j.1538-4632.1992.tb00261.x

[52] R Core Team. A language and environment for statistical computing. Vienna, Austria: R Foundation for Statistical Computing. 2017. http://www.R-project.org

[53] Pebesma E. Simple features for R: standardized support for spatial vector data. R J. 2018;10(1):439–46. doi: 10.32614/RJ-2018-009

[54] Bivand RS, Wong DWS. Comparing implementations of global and local indicators of spatial association. TEST. 2018;27(3):716–48. doi: 10.1007/s11749-018-0599-x

[55] Dunnington D. ggspatial: Spatial Data Framework for ggplot2. 2025.

[56] Walker K. Tigris: An R package to access and work with geographic data from the US Census Bureau. https://digitalcommons.unl.edu/r-journal/491/. 2016. Accessed 2026 August 28.

[57] Stapp P, Mc Kenzie A, Bucklin DM, Baldwin RA, Quinn N. Patterns of exposure of coyotes to anticoagulant rodenticides in California, USA. J Wildl Manag. 2025;89(2):e22696. doi: 10.1002/jwmg.22696

[58] Gabriel MW, Woods LW, Poppenga R, Sweitzer RA, Thompson C, Matthews SM, et al. Anticoagulant rodenticides on our public and community lands: spatial distribution of exposure and poisoning of a rare forest carnivore. PLoS One. 2012;7(7):e40163. doi: 10.1371/journal.pone.0040163 22808110 PMC3396649

[59] Grand View Research. Anticoagulant rodenticides market size, share & trends analysis report by product type (1st generation, 2nd generation), by form (pellets, blocks, powders), by application, by region, and segment forecasts, 2022–2030. 2021. https://www.grandviewresearch.com/industry-analysis/anticoagulant-rodenticides-market-report

[60] Damin-Pernik M, Espana B, Besse S, Fourel I, Caruel H, Popowycz F. Development of an ecofriendly anticoagulant rodenticide based on the stereochemistry of difenacoum. Drug Metab Dispos. 2016;44(12):1872–80. doi: 10.1124/dmd.116.071688 27621204

[61] Fisher P, Brown S, Arrow J. Pindone residues in rabbit tissues: Implications for secondary hazard and risk to non-target wildlife. Wildlife Res. 2015;42(4):362–70. doi: 10.1071/WR15019

[62] Serieys LEK, Foley J, Owens S, Woods L, Boydston EE, Lyren LM, et al. Serum chemistry, hematologic, and post-mortem findings in free-ranging bobcats (Lynx rufus) with notoedric mange. Journal of Parasitology. 2013;99(6):989–96. doi: 10.1645/12-175.1 23957865

[63] Sussman AL, Gardner B, Adams EM, Salas L, Kenow KP, Luukkonen DR, et al. A comparative analysis of common methods to identify waterbird hotspots. Methods Ecol Evol. 2019;10(9):1454–68. doi: 10.1111/2041-210x.13209

[64] Lin G. Comparing spatial clustering tests based on rare to common spatial events. Computers, Environment and Urban Systems. 2004;28(6):691–9. doi: 10.1016/j.compenvurbsys.2003.07.002

[65] Kontos CC, Bologna PAX. Extirpation and reappearance of the fisher (Martes pennanti) in New Jersey. Bull NJ Acad Sci. 2023;53(3):1–4.

[66] Morzillo AT, Schwartz MD. Landscape characteristics affect animal control by urban residents. Ecosphere. 2011;2(11):art128. doi: 10.1890/es11-00120.1

[67] Hindmarch S, Elliott J, Weir R, Lee S, Maisonneuve F. Anticoagulant rodenticide exposure in endangered American badgers (Taxidea taxus) and fishers (Pekania pennanti) from British Columbia, Canada, 1998 to 2018. Environ Pollut. 2025;375:126278. doi: 10.1016/j.envpol.2025.126278 40252754

[68] Elliott JE, Silverthorn V, Hindmarch S, Lee S, Bowes V, Redford T. Anticoagulant rodenticide contamination of terrestrial birds of prey from western Canada: patterns and trends, 1988–2018. Environmental Toxicology and Chemistry. 2022;41(8):1903–17. doi: 10.1002/etc.5361 PMC9540899 35678209

[69] Murray M. Anticoagulant rodenticide exposure and toxicosis in four species of birds of prey in Massachusetts, USA, 2012–2016, in relation to use of rodenticides by pest management professionals. Ecotoxicol. 2017;26(8):1041–50. doi: 10.1007/s10646-017-1832-1 28669046

[70] Bartos M, Dao S, Douk D, Falzone S, Gumerlock E, Hoekstra S, et al. Use of anticoagulant rodenticides in single-family neighborhoods along an urban-wildland interface in California. CATE. 2012;4(1). doi: 10.15365/1932-7048.1100

[71] Sainsbury KA, Shore RF, Schofield H, Croose E, Pereira MG, Sleep D, et al. Long-term increase in secondary exposure to anticoagulant rodenticides in European polecats Mustela putorius in Great Britain. Environ Pollut. 2018;236:689–98. doi: 10.1016/j.envpol.2018.02.004 29438955

[72] Buckley JY, Murray MH, de la Sancha NU, Fidino M, Byers KA, Fyffe R, et al. Widespread exposure to anticoagulant rodenticides among common urban mesopredators in Chicago. Sci Total Environ. 2024;952:175883. doi: 10.1016/j.scitotenv.2024.175883 39222805

[73] Regnery J, Snelinski B, Bachtin J, Möhlenkamp C, Schmolz E, Friesen A. Indirect contamination of cockroaches by anticoagulant rodenticides. Environmental Chemistry Letters. 2025;23(3):759–64. doi: 10.1007/s10311-025-01821-y

[74] Donovan TM, Freeman M, Abouelezz H, Royar K, Howard A, Mickey R. Quantifying home range habitat requirements for bobcats (Lynx rufus) in Vermont, USA. Biol Conserv. 2011;144(12):2799–809. doi: 10.1016/j.biocon.2011.06.026

[75] Nusser SM, Clark WR, Otis DL, Huang L. Sampling considerations for disease surveillance in wildlife populations. J Wildl Manag. 2008;72(1):52–60. doi: 10.2193/2007-317

